# Anaerobe-associated microbial shifts at infection onset in diabetes-related foot ulcers revealed by longitudinal metagenomics

**DOI:** 10.3389/fcimb.2026.1812721

**Published:** 2026-06-02

**Authors:** Michael Radzieta, Matthew Malone, Saskia Schwarzer, Emma Bergamin, Greg Whitely, Slade Jensen

**Affiliations:** 1 Infectious Diseases and Microbiology, School of Medicine, Western Sydney University, Sydney, NSW, Australia; 2 Ingham Institute for Applied Medical Research, Sydney, NSW, Australia; 3 South West Sydney Limb Preservation and Wound Research, South Western Sydney Local Health District, Liverpool Hospital, Sydney, NSW, Australia; 4 High Risk Foot Service, Liverpool Hospital, South Western Sydney Local Health District, Liverpool, NSW, Australia; 5 Whitely Corporation, Tomago, NSW, Australia

**Keywords:** diabetes mellitus, diabetic foot ulcer, infection, microbiome, wounds

## Abstract

**Introduction:**

Diabetes-related foot infections (DRFIs) are a major cause of hospitalisation and carry a significantly increased risk of lower extremity amputation. To date there is a lack of longitudinal studies examining within-patient microbiome dynamics during the transition from non-infected to infected diabetes-related foot ulcers (DRFUs).

**Methods:**

We used shotgun metagenomic sequencing to longitudinally profile the wound microbiome of 6 patients with DRFUs who developed clinical infections, utilising taxonomic profiling, metagenome assembly and binning and strain level analysis to characterise within-patient microbial shifts.

**Results:**

DRFUs with no signs of clinical infection were colonised by virulent pathogens including *Staphylococcus aureus, Streptococcus agalactiae, Enterococcus faecalis, Enterobacter hormaechei* and *Pseudomonas aeruginosa*. In most patients, infection onset was associated with a decrease in pathogen abundance and a significant increase in obligate anaerobes including *Prevotella* spp, *Peptoniphilus* spp, *Porphyromonas* spp and *Anaerococcus* spp.

**Conclusion:**

These findings highlight the potential importance of anaerobes and hypoxia in DRFIs and may support monitoring of tissue oxygen saturation as a predictor of infection onset.

## Introduction

Diabetes-related foot ulcers (DRFUs) are clinically challenging, with a recent global meta-analysis of 32 randomized controlled trials demonstrating poor healing rates with only 33% of ulcers healing within a 12–24-week window under standard of care interventions (Coye et al., 2025). Several comorbidities associated with diabetes including peripheral neuropathy (PN), peripheral arterial disease (PAD) and altered foot structure as major drivers in the formation of DRFUs, which will affect 19% to 34% of people with diabetes in their lifetime (McDermott et al., 2022). Individuals with diabetes have several ill-defined immunological disturbances which may account for the increased risk of developing infection, which occurs in approximately 50% to 60% of ulcers, with 20% of moderate to severe infections resulting in lower extremity amputations (Berbudi et al., 2020; Edmonds et al., 2021). Diagnosis of an infected DRFU currently relies on clinical examination, supported by a relevant history of the event and supporting pathology testing. To complicate the clinical picture, the typical signs of infection can often be absent, with these masked symptoms attributed to the effects of immune disturbances, PN and PAD which impacts accurate detection (Senneville et al., 2023).

There has been increasing interest in understanding the role of the microbiome on clinical outcomes with numerous studies being published over the last 10 years (Gardiner et al., 2017; Gardner et al., 2013; Kalan et al., 2019; Malone et al., 2022; Radzieta et al., 2022, Radzieta et al., 2021; Saeb et al., 2021; Travis et al., 2020). The adoption of next generation sequencing (NGS) techniques has been critical in revealing previously unknown dynamics of the DRFU microbiome including the ubiquity of anaerobes within wounds, significant patient to patient heterogeneity and the complex polymicrobial nature of wounds (Liu et al., 2020). While anaerobes are now frequently identified in microbiome studies, their role and impact on the wound microenvironment remains poorly understood outside of few studies which have identified and association between anaerobe abundance and poor wound outcomes (Coluccio et al., 2024). There are relatively few studies on the microbiome of diabetes-related foot infections (DRFI). Cross-sectional studies of DRFIs have revealed highly diverse communities often dominated by species including *Staphylococcus aureus*, *Streptococcus* spp, *Pseudomonas aeruginosa* and Enterobacteriaceae (Macdonald et al., 2021; Malone et al., 2017; Mudrik-Zohar et al., 2022). However, these studies provide only a snapshot of infection at the time of sampling and do not capture within patient changes that facilitate the transition from a non-infected wound to clinically infected. Longitudinal studies, such as those by Gardiner et al. and Kalan et al. have begun to address this gap by following DRFUs over time (Gardiner et al., 2017; Kalan et al., 2019). Gardiner et al. tracked changes in the wound microbiome of 8 patients over a 10-week period and identified a dynamic microbiome that changed over time within patients. Furthermore, they highlighted the abundance of anaerobic species such as *Finegoldia* that have frequently been observed in wound microbiome studies utilizing NGS techniques (Gardiner et al., 2017; Macdonald et al., 2021; Malone et al., 2017). Kalan et al. published a major study that tracked 100 patients for up to 26 weeks with sample collected subject to metagenomic sequencing. Results from this study identified that strain level variation in *S. aureus* and genetic signatures of biofilm were associated with poor outcomes (Kalan et al., 2019). While these studies highlight the importance of within patient longitudinal sampling, it is critical to note that no longitudinal studies have been published that track changes within patients as they progress from a non-infected to infected state.

In this pilot study, we utilized shotgun metagenomic sequencing to explore longitudinal samples collected from patients with DRFUs, to examine the microbiome dynamics of patients who developed a DRFI. By analyzing these samples at high resolution, we sought to identify microbial shifts, including strain dynamics that are associated with the transition to infection. This approach allowed for the identification of within patient changes over time, providing insights into the temporal dynamics of microbial communities that cannot be captured by cross-sectional studies of singular sample timepoints. This study represents the first longitudinal investigation of DRFI microbiomes, with the potential to inform strategies for early diagnosis, targeted treatment and improved clinical management.

## Methods

### Study design and sampling

The collection of samples was undertaken as part of a larger project aimed at identifying biomarkers within DRFUs, with a sub-analysis to identify patients who developed infection during the course of their DRFU management. Ethics approval for this study was granted by the South West Sydney Local Health District Research and Ethics Committee (2020/ETH02409), with all participants providing written informed consent prior to inclusion. Over a 24-month period, participants with DRFUs were prospectively enrolled from the Liverpool Hospital High Risk Foot Service with a DRFU occurring below the malleolus. Ulcers were sampled longitudinally starting from baseline (Week 0), followed by weeks 1, 3, 6, 12, 24, 36, 48, 52, or until the ulcer had healed, with additional samples collected at sentinel events. All patients received standard wound care throughout the study period in accordance with clinical management protocols at the Liverpool Hospital High Risk Foot Service, including cleansing with saline, regular sharp debridement of the wound bed and periwound, pressure offloading and dressing with non-medicated wound dressings based on exudate levels. Systemic antibiotic therapy was initiated at the point of infection diagnosis as detailed in **Table 1**.

**Table 1:** Clinical metadata for the six patients included in this study. Patients are presented with relevant demographic, clinical and microbiological data collected at each sampling timepoint across the study period.

Patient	Sex	Age	Diabetes	Diabetes medication	Sample	PEDIS^1^	WIfI ulcer grade^2^	Wound progress	Wound duration (weeks)	Antibiotic therapy prescribed^3^	Wound swabs^4^
P1	F	65	Type II	Insulin + DPP4i	Baseline	1	1	Non-infected but stalled wound	104	–	–
					Infection	3	2	Infected	108	Augmentin Duo Forte changed to IV Tazocin as poor clinical response.	3+ mixed coliforms 3+ mixed skin flora
					Resolution	1	1	Infection resolved and healing	110	Augmentin Duo Forte	–
P2	F	69	Type II	Metformin + DPP4i + SGLT2i	Baseline	2	1	Infected	8	Oral Cephalexin	1+ mixed coliforms 1+ mixed skin flora
					Infection Progression	3	1	Infected	9	Augmentin Duo Forte	1+ *S. aureus* 1+ mixed skin flora
					Infection Downgraded	2	1	Infected	10	Augmentin Duo Forte	–
P3	M	51	Type II	Insulin	Baseline	1	1	Non-infected wound	32	–	–
					Infection	3	2	Infected	35	Flucloxacillin	2+ *S. aureus* 2+ *M. morganii* 1+ *A. faecalis*
					Infection Downgraded	2	1	Infected	37	Flucloxacillin	–
P4	M	49	Type II	Insulin	Baseline	1	1	Healing	180	–	–
					Infection	4	3	Infected	208	Flucloxacillin + Ciprofloxacin	2+ Group B Streptococcus 1+ *P. aeruginosa* 1+ mixed skin flora
P5	M	52	Type I	Insulin	Baseline	1	1	Healing	24	–	–
					Infection	4	3	Infected	54	Self-started Augmentin Duo Forte changed to IV Tazocin	3+ *E. faecalis* 1+ mixed skin flora
					Resolution	1	1	Healing	57	–	–
P6	M	51	Type I	Insulin	Baseline	1	1	Non-infected but wound stalled	5	–	–
					Infection	2	1	Infected	7	IV Cefazolin and oral Flucloxacillin + Metronidazole	3+ *S. aureus* 2+ mixed skin flora
					Resolution	1	1	Healing	8	Oral Flucloxacillin + Metronidazole	–

^1^ PEDIS rating determined based on IWGDF guidelines. PEDIS 1, non-infected; PEDIS 2, mild infection; PEDIS 3, moderate infection; PEDIS 4, Severe infection.

^2^ WIfI ulcer grade. 1, Small, shallow ulcer; 2, Deep ulcer; 3, Extensive, deep ulcer.

^3^ The antibiotics outlined are indicative of those prescribed upon infection diagnosis when the sample was collected unless stated otherwise (P2 and P5).

^4^ Wound swab culture results are reported using semiquantitative scoring based on the four quadrant streak method: 1+, growth in quadrant 1 only (sparse); 2+, growth in quadrants 1 and 2 (moderate); 3+, growth in quadrants 1, 2 and 3 (heavy, approximately 100 colonies).

A sentinel event was defined as an event requiring additional sampling outside of the standard timepoints. These events were; (1) wounds achieving a >80% wound area reduction from baseline. The rationale for choosing >80% wound area reduction as a sentinel event for specimen collection was based on assumptions that participants could fully heal or achieve 100% epithelialization before returning to the next scheduled timepoint; (2) development of a clinical infection and when the infection was clinically deemed to be resolved or treatment failed.

The diagnosis and severity of infection was based on clinical observations as defined by the International Working Group for the Diabetic Foot (IWGDF), Diabetic Foot Infection Guidelines (Senneville et al., 2023). Infection severity was classified using PEDIS grades (PEDIS 1 – no infection, PEDIS 2 – mild infection, PEDIS 3 – moderate infection, PEDIS 4 – severe infection). Wound swabs were collected following debridement and cleansing with saline using the Levine technique for wound sampling before being placed in DNA/RNA shield (Zymo Research) and stored at -80°C.

### DNA extraction and sequencing

Frozen swabs were thawed on ice and DNA was extracted using the ZymoBIOMICS DNA Miniprep kit, according to the manufacturer’s instructions. Host DNA was then depleted using the NEBNext Microbiome DNA Enrichment Kit, according to the manufacturer’s instructions. Enriched DNA was then submitted to the Ramaciotti Centre for Genomics (Sydney, AUS) for library generation using the Illumina DNA Prep library kit and sequencing using a NovaSeq 6000 platform on a S4 flowcell (2x150bp).

### Bioinformatic analysis

Sequencing reads were processed with Kneaddata (https://github.com/biobakery/kneaddata) for quality control and host DNA removal using default parameters. After host removal, remaining reads were analyzed using Metaphlan3 for taxonomic assignment and estimation of relative abundances using default parameters (https://github.com/biobakery/MetaPhlAn). Individual samples were assembled using Spades with default parameters in addition to the --meta flag being enabled (https://github.com/ablab/spades). Genome binning was then completed using Vamb (https://github.com/RasmussenLab/vamb) with default parameters and the --minfasta flag enabled, with 1,000,000 bp specified as the minimum bin length. Bin quality was assessed using CheckM with default parameters (https://github.com/Ecogenomics/CheckM) followed by taxonomic assignment using GTDB-TK (https://github.com/Ecogenomics/GTDBTk) with default parameters. Finally, high quality bins were annotated using BV-BRC (https://www.bv-brc.org) against the VFDB and CARD databases for virulence and antibiotic resistance gene prediction, respectively. StrainGE was used for strain prediction (https://github.com/broadinstitute/StrainGE) with custom databases built for species of interest based on the developers’ instructions. StrainGST was run first on kmerized host depleted reads for strain prediction followed by StrainGR for estimating strain similarity across patient samples.

### Statistical analysis

Statistical analyses were performed in R (v4.5.1). Diversity analyses were conducted on species level taxonomic profiles using the vegan package (v2.7). Bray Curtis dissimilarity matrices were generated for principal coordinate analysis (PCoA) to assess beta diversity, while alpha diversity was assessed using the Richness, Shannon and Simpson indices. Differences in beta diversity between infection states was assessed using PERMANOVA (adonis2 function). Changes in microbial abundance were assessed using linear mixed-effects models (LMMs) implemented in lme4 (v1.1.37) with lmerTest (v3.1.3) for significance testing. Patient identity was included as a random intercept to account for repeated measures within individuals. Results were considered significant if p.value < 0.05. Taxonomic profiles generated by MetaPhlAn3 were imported into R and filtered to genus or species level classifications directly from the merged taxonomic profile output file. Obligate anaerobic genera were identified by examining the most abundant taxa across all samples and classifying those with known obligate anaerobic physiology based on established literature (Coluccio et al., 2024; Uberoi et al., 2024). Total obligate anaerobe abundance per sample was calculated by summing the relative abundances of all identified obligate anaerobic genera. In all figures where PEDIS grade is displayed on the x-axis, values are ordered chronologically from left to right representing sequential sampling timepoints for each patient. Where the same PEDIS grade appears multiple times within a patient panel this reflects distinct sampling timepoints at which the same infection severity was recorded.

## Results

### Study cohort

Following analysis of the broader study information, we identified 5 patients who transitioned from an un-infected DRFU to a clinically infected DRFU, and 1 patient who experienced a worsening infection, transitioning from a mild (PEDIS 2) to moderate (PEDIS 3) skin and soft tissue infection. The cohort comprised 4 males and 2 females with a median age of 51.5 years (range 49-69). Four patients had Type II diabetes and 2 had Type I diabetes. All patients were receiving insulin or oral hypoglycemic agents for diabetes management, with 2 patients receiving adjunct DPP4 inhibitor therapy and 1 patient receiving Metformin and SGLT2 inhibitor therapy. Wound duration at baseline ranged from 5 to 180 weeks, reflecting the chronic nature of DRFUs in this cohort. All 6 patients received systemic antibiotic therapy during their management of infection (**Table 1**) and all were sampled according to the same longitudinal protocol as the broader study, with samples collected at baseline (Week 0) and subsequent scheduled timepoints, with additional samples collected at sentinel events including infection onset and resolution. Patients are labeled as P1-P6.

### Metagenomics reveals shifts in the longitudinal microbiome associated with infection onset and resolution

Shotgun metagenomic sequencing produced a median of 310,122,786 read pairs per sample (103,387,052 to 612,329,670 read pairs) which were processed using KneadData for host removal. This produced a median of 1,335,067 non-human read pairs per sample (773,284 to 65,115,004 read pairs) for taxonomic profiling using Metaphlan3, with the microbial content ranging from 0.14% to 19.7% of the total sequencing output despite host depletion with the NEBNext Enrichment kit (**Supplementary Figure 1**).

**Figure 1:**
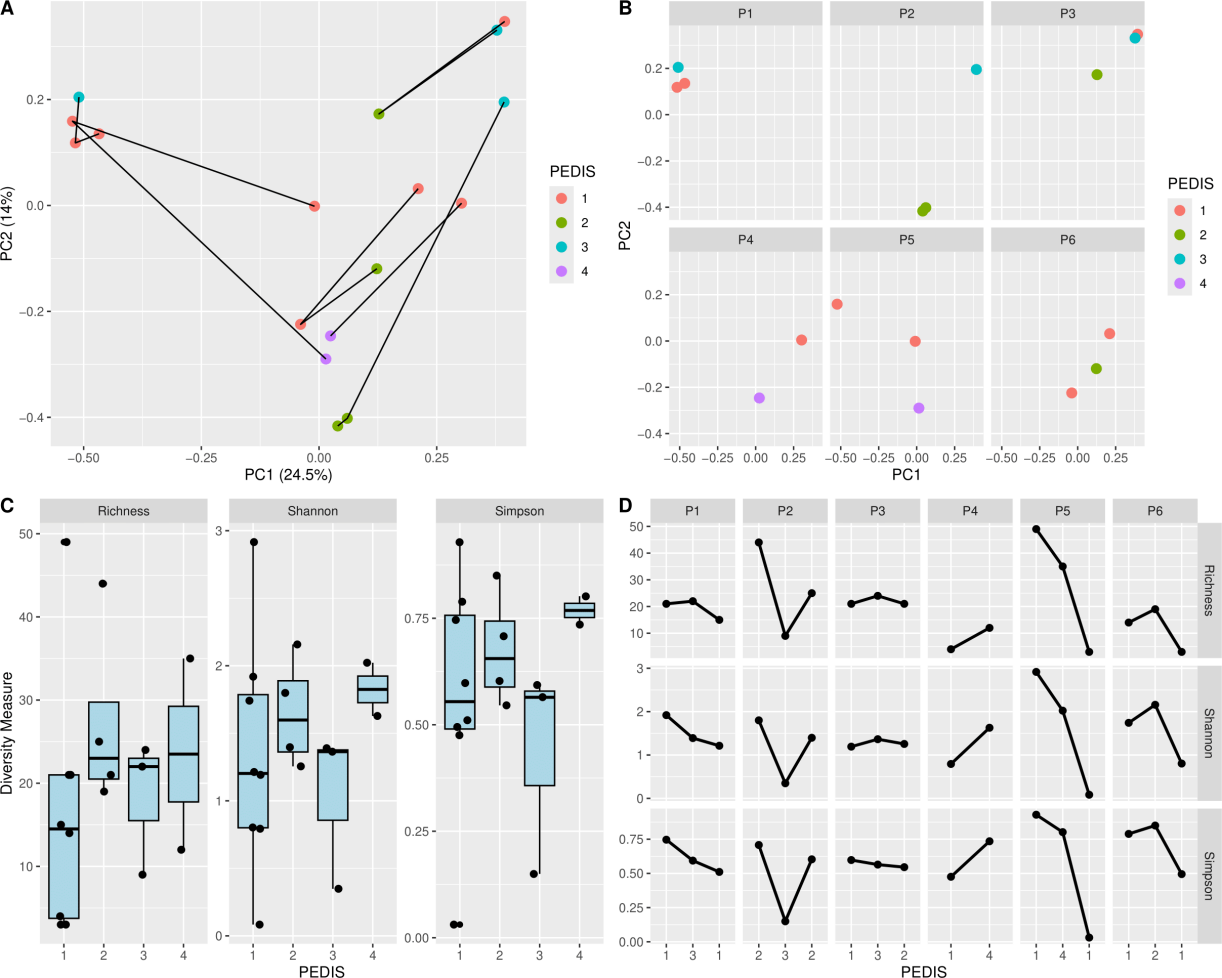
Microbial diversity. **(A)** PCoA analysis stratified by PEDIS classification with lines linking samples taken from each patient. **(B)** Faceted PCoA by patient with sample stratified by PEDIS classification. **(C)** Alpha diversity measures stratified by PEDIS classification. **(D)** Temporal shifts in alpha diversity measures within patients. The x-axis represents the PEDIS grade recorded at each sequential sampling timepoint, ordered chronologically from left to right. Connected points represent successive samples from the same patient, illustrating changes in diversity as patients transition between non-infected (PEDIS 1) and infected states (PEDIS 2-4). Note that where the same PEDIS grade appears multiple times within a patient this reflects distinct sampling timepoints at which the same infection severity was recorded.

To understand how the microbial composition changed with infection onset, we first characterized overall community structure before examining within-patient diversity. We began by assessing beta diversity (difference in microbial community composition between samples), followed by alpha diversity (within sample diversity) and subsequent taxonomic analysis. Beta diversity was assessed using the Bray-Curtis index on species classifications to evaluate differences in community composition between samples. PERMANOVA analysis using adonis2 identified a significant effect of patient identity on community composition (R^2^ = 0.54, p.value = 0.001***) suggesting that microbial profiles were highly patient specific. Therefore, subsequent analysis of infection severity (PEDIS) was performed using permutations constrained within patients to control for repeated measures. Using this model, we identified that infection severity was significantly associated with microbial community composition (R^2^ = 0.22, p.value = 0.01*) which is observable in Principal Coordinate Analysis (PCA) (**Figures 1A, B**).

Due to microbial profiles being highly patient specific, we then assessed how microbial communities diverged from baseline samples of non-infected to infected (or increasing infection severity) by taking a within-patient approach. Using the Bray-Curtis dissimilarity across all patients, the progression from a baseline PEDIS 1 (P1, P3, P4, P5, P6) or PEDIS 2 (P2) to higher infection severities was associated with an increase in microbial community dissimilarity, ranging from 0.25 to 0.95 (**Supplementary Figure 2**). In 3/6 patients the dissimilarity relative to baseline increased upon both infection resolution or reduction of infection severity, while in 2/6 patients the dissimilarity decreased toward baseline. There was no follow up sample collected for 1 patient (P4).

Alpha diversity was assessed using the Simpson (dominance of most abundant species), Shannon (species number and distribution) and Richness (species number) metrics on samples grouped by PEDIS grade (**Figure 1C**) and longitudinally within patients (**Figure 1D**). There were no significant differences in the three metrics measured between PEDIS grades combined across patients. When looking at shifts in alpha diversity within patients longitudinally, we observed an increase in alpha diversity associated with infection onset and subsequent decrease with infection resolution in 3/6 patients. In 2/3 remaining patients we observed a decrease in alpha diversity associated with infection onset and in the final patient alpha diversity remained relatively constant.

**Figure 2:**
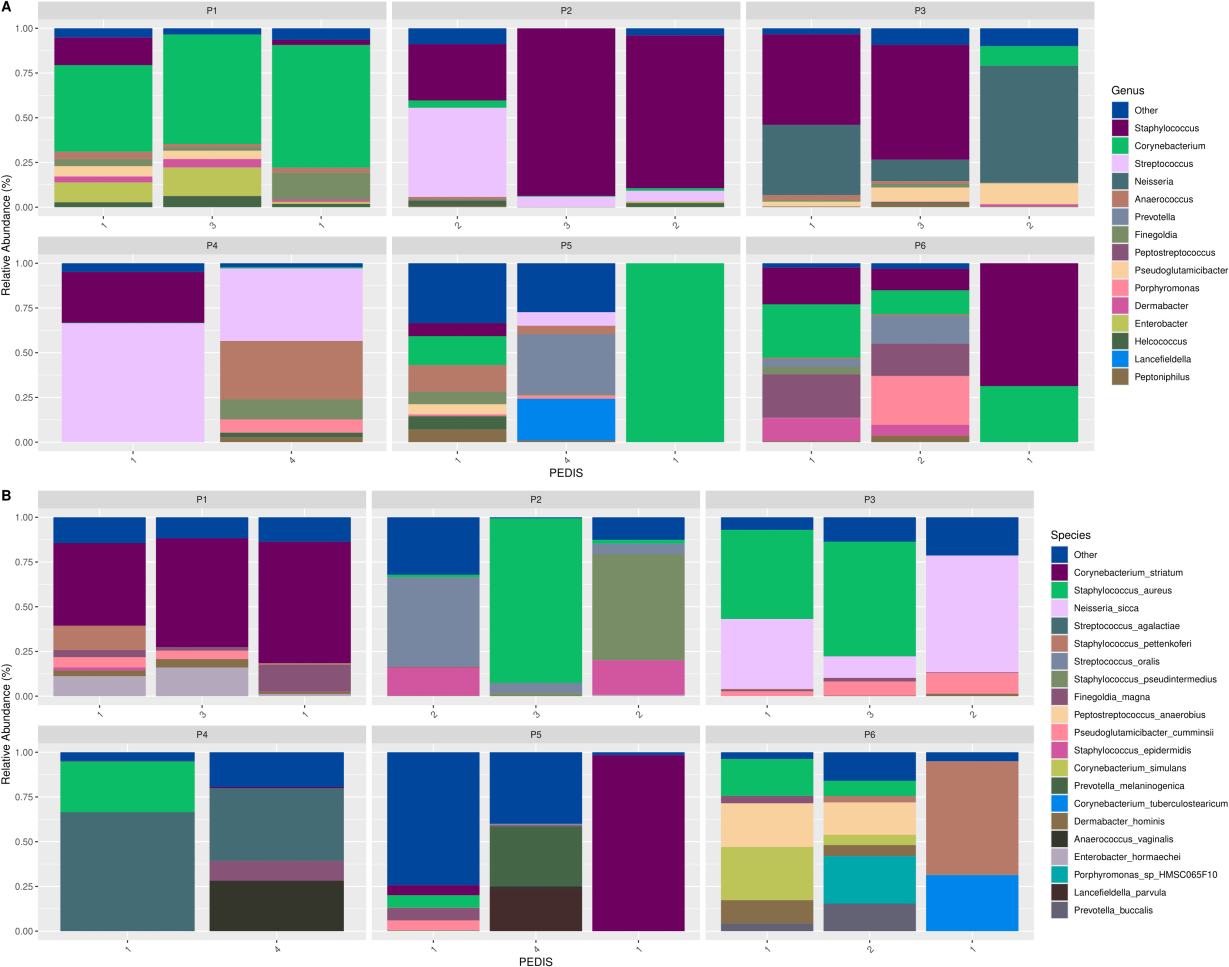
Taxonomic bar charts of the most dominant bacteria identified by metaphlan3. **(A)** Taxa stratified to the genus level. **(B)** Taxa stratified to the species level. Taxa representing <5% abundance across all patients were condensed to “other” to show the most abundance taxa.

Taxonomic analysis identified unique microbiome profiles within each patient which changed as DRFUs transitioned between non-infected and infected pathologies. Microbial classifications were stratified at the genus and species level and identified common pathogens associated with DFIs including *Staphylococcus aureus* and *Streptococcus agalactiae* (**Figure 2**). Infection in P1 was associated with a shift in a higher abundance of *Enterobacter hormaechei* at baseline non-infected to infected. Subsequently, the abundance of *E. hormaechei* decreased at infection resolution. In P2 and P3, increasing infection severity and transition from non-infected to infected, respectively, was associated with higher abundances of *S. aureus*. In P4 we observed a high abundance of *S. agalactiae* and *S. aureus* in the non-infected baseline samples, which decreased in abundance upon infection onset. At the same time, we observed an increased abundance of obligate anaerobes including *Finegoldia magna* and *Anaerococcus vaginalis*. Similarly, in P5 we observed a shift from non-infected baseline with an increased abundance of obligate anaerobes during infection including *Lancefieldella paruvula* and *Prevotella melaninogenica.* In conjunction with this, there was a decrease in the abundance of known pathogens including *S. aureus* that were present at baseline. Finally, in P6 we observed that infection onset was associated with an increased abundance of obligate anaerobes including *Porphyromonas* spp, *Prevotella* spp and *Peptostrepcoccus* spp which accounted for >50% of the total microbiome. Concurrently, the abundance of *S. aureus*, *Corynebacterium simulans* and *Dermabacter hominis* decreased.

**Figure 3:**
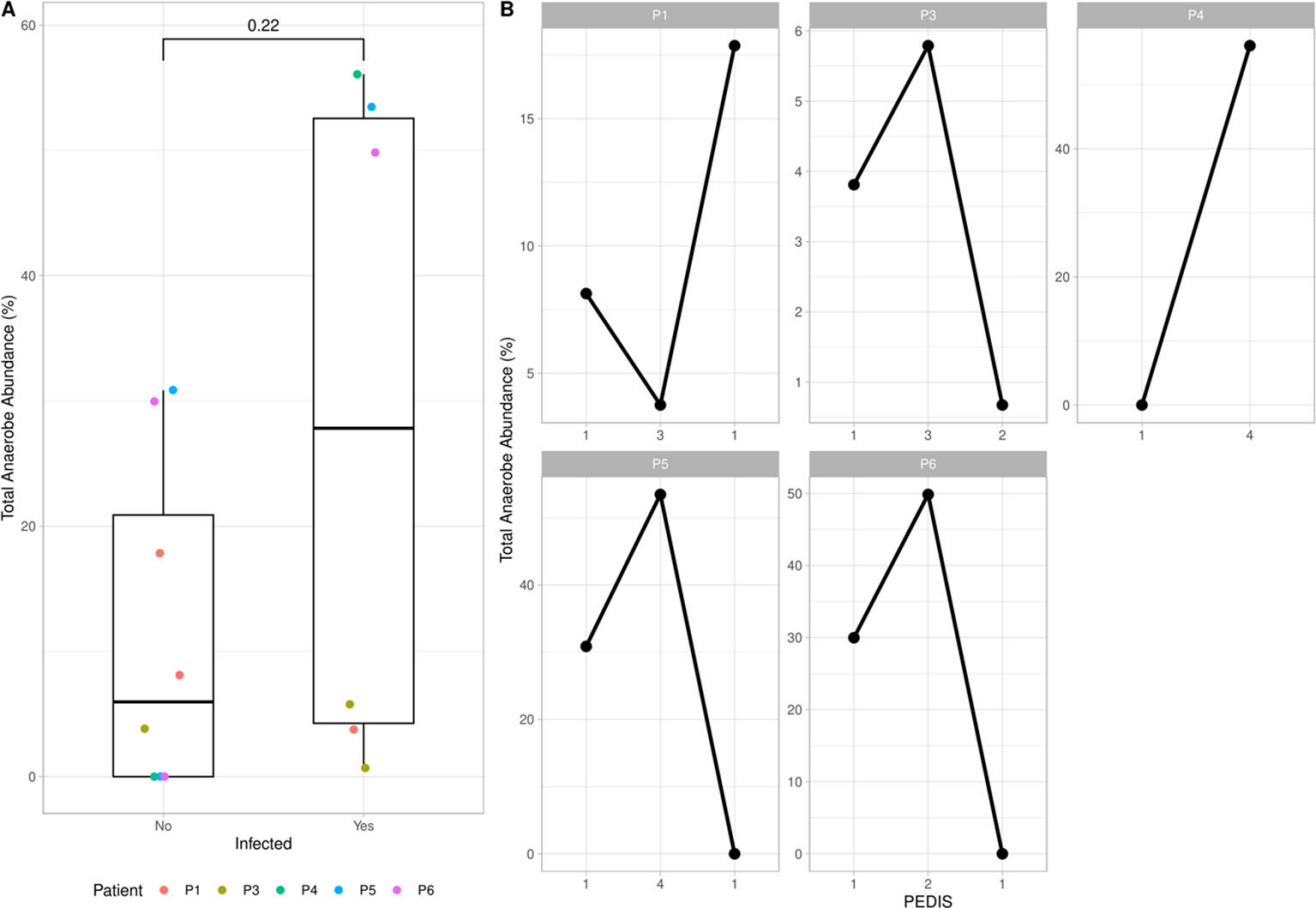
Abundance of obligate anaerobes. **(A)** Comparison of the overall abundance of obligate anaerobes between infected and non-infected wounds. Non-infected wounds includes baseline and infection resolution samples. **(B)** Temporal changes in obligate anaerobe abundance within each patient. P2 was not included in this analysis due to missing a before infection sample.

Based on these observations we then examined the abundance of anaerobic species after identifying highly abundant anaerobic taxa that presented during the shift from non-infected to infected samples including *Anaerococcus* spp, *Finegoldia* spp, *Helcococcuss* spp, *Peptostreptococcuss* spp, *Porphyromonas* spp, *Veillonella* spp, *Prevotella* spp and *Lancefieldella* spp. The total abundance of anaerobic genus was determined and compared between infected and non-infected wounds (**Figure 3A**). A linear mixed model which accounted for within patient differences estimated that infected wounds had an average anaerobe abundance of 84.4% compared to 21.5% in non-infected wounds (p.value = 0.0556). Analysis of within patient shifts over time identified an increase in anaerobe abundance in P3, P4, P5 and P6 (**Figure 3B**). P1 remained the only outlier with a decrease in anaerobe abundance upon infection onset.

### Genome binning identifies pathogenic species associated with infection

Individual samples were then assembled using SPAdes with the --meta flag to enable the metagenome assembly mode. Assemblies from each patient timepoint were then concatenated and the genomes were binned using Vamb with default parameters. Bin quality was then assessed using CheckM and taxonomically assigned using GTDB-Tk, with high quality (HQ) bins (>90% completeness, <5% contamination) then being annotated using BV-BRC to identify the presence of virulence factors (VFs) and antibiotic resistance genes (ARGs). Likely due to fewer microbial reads within P6 samples, no bins were extracted, and they were removed from any further analysis.

**Figure 4:**
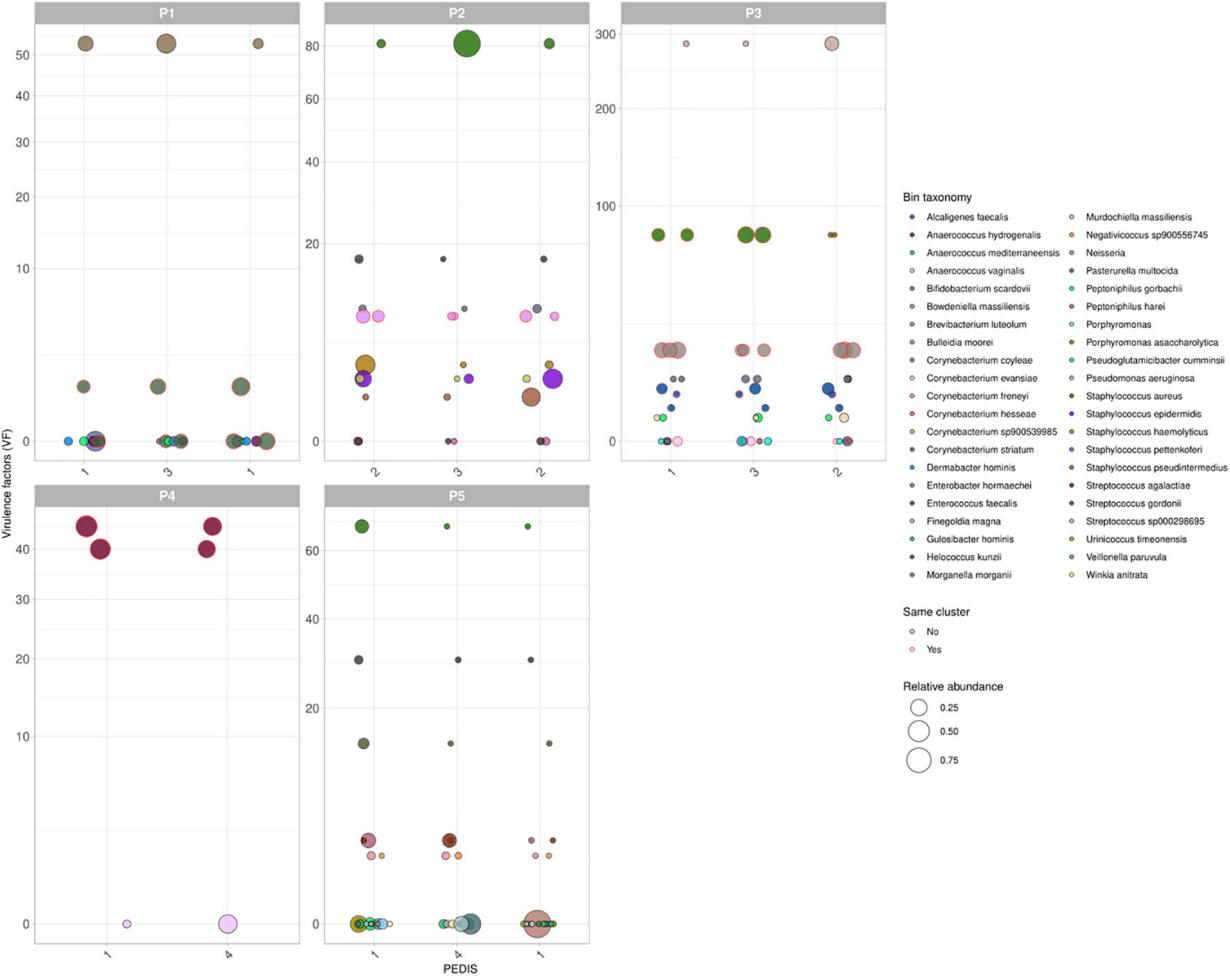
Binned genomes identified within each patient. Relative abundance of each bin is visualized by the size of each bubble with bins estimated to be from the same cluster based on Vamb output are outlined red. The y-axis shows the number of virulence genes each bin carries following annotation to the VFDB using BV-BRC.

In total 69 high quality bins were assembled across 5 patients (**Supplementary Data Sheet 1**). Bin relative abundances were estimated by mapping reads to contigs with Strobealign/AEMB, assigning contigs to bins based on VAMB’s vae_clusters_split output and summing coverage per bin. Finally, the coverage was normalized to the total coverage per sample (**Figure 4**).

Eight HQ bins were identified in P1 with only 2 carrying virulence genes. Notably, an *E. hormaechei* bin increased from an estimated abundance of 19.5% to 39% upon infection onset then reduced to 0.5% upon infection resolution. Genome annotation identified this bin was carrying 29 VF and 57 ARGs, including gene clusters associated with iron acquisition (entA/E/S, fepB/C/D/G and iroB/C/D/N), curli production (csgD/E/F/G), motility and chemotaxis (fliM/G/P, flgB/C/G/H, flhA, motA, cheW, cheY), haem uptake (chuA/S) and LPS production. In the treatment of this patient, an initial empiric antibiotic therapy of Augmentin Duo Forte (Amoxicillin/clavulanate 875 mg/125 mg) failed and therapy was escalated to intravenous Tazocin (Totalling 13.5 g [12 g piperacillin/1.5 g tazobactam), which may be explained by the presence of an AmpC beta-lactamase (ACT-32) identified within the *E. hormaechei* genome.

In P2, 11 HQ bins were identified with a *S. aureus* bin being the most abundant when infection increased in severity from PEDIS 2 to PEDIS 3 (2.1% > 93% abundance). Genome annotations identified a highly virulent bin carrying 68 VF and 17 ARGs, including genes associated with adhesion (icaA/B/C/D/R, sdrC/D/E, clfA/B, fnbA, spa, ebp, map), immune evasion (scn, sbi, sak, vWbp, adsA), capsule biosynthesis (cap8A-P), toxin production (hlY/A/B/D, hlgA/B/C, lukF-PV, seb), exoprotein production (sspA/B/C, aur, geh, lip, hysA, clpP), iron acquisition (isdA/B/C/D/E/F/G, srtB) and the Type VII secretion system (essA/B/C, esaA/B/C, esxA/B).

Similarly, transition from non-infected to infected DFU in P3 was associated with an increase in abundance of a *S. aureus* bin carrying 60 VF and 18 ARGs, 59 of which were identical to those found in the *S. aureus* bin from P2. A second bin in P3 identified as *Neisseria* spp was also assembled which carried 11 VF and 9 ARGs which were associated with iron acquisition (hmbR), adhesion (pilT/T2/B, luxS), efflux (farB, mtrC/D), oxidative stress defense (katA, mntC) and DNA repair (recN). Upon infection downgrade from PEDIS 3 to PEDIS 2 following treatment with flucloxacillin, a *P. aeruginosa* bin increased in abundance from <1% to 15% which carried >250 VF associated with multiple secretion systems (Type I, Type II, Type III, Type VI), motility, biofilm production, toxins, iron uptake, siderophore production and regulation.

Only 3 high quality bins were assembled from P4 with the most abundant being a *S. agalactiae* cluster carrying approximately 45 VF and 5 ARGs. VF present on the genome include genes associated with hemolysin/cytolysin production (cylA/B/D/E/F/G/I/J/K/X/Z), capsule synthesis (hasC), sialic acid synthesis (neuA/B/C), adhesins (fbsA, lmb, scpA/B, bca, pilA/C, srtC1/C2/C3/C4) and secreted factors (cfA/B, hylB, acpC). The estimated abundance of the *S. agalactiae* cluster decreased from approximately 90% to 60% upon infection onset along with an increase in abundance of an *A. vaginalis* (14% to 35%) bin carrying 0 VF.

Finally, 29 high quality bins were assembled from P5. Several binned genomes carried >10 VF including *S. aureus*, *E. faecalis* and *P. multocida*. All three bins decreased in estimated abundance (<1%) as the wound progressed to a severe infection, with several bins increasing in abundance including *S. gordonii* and two anaerobic species (*B. scardovvi* and *M. massiliensis*).

### Strain dynamics associated with infection severity

**Figure 5:**
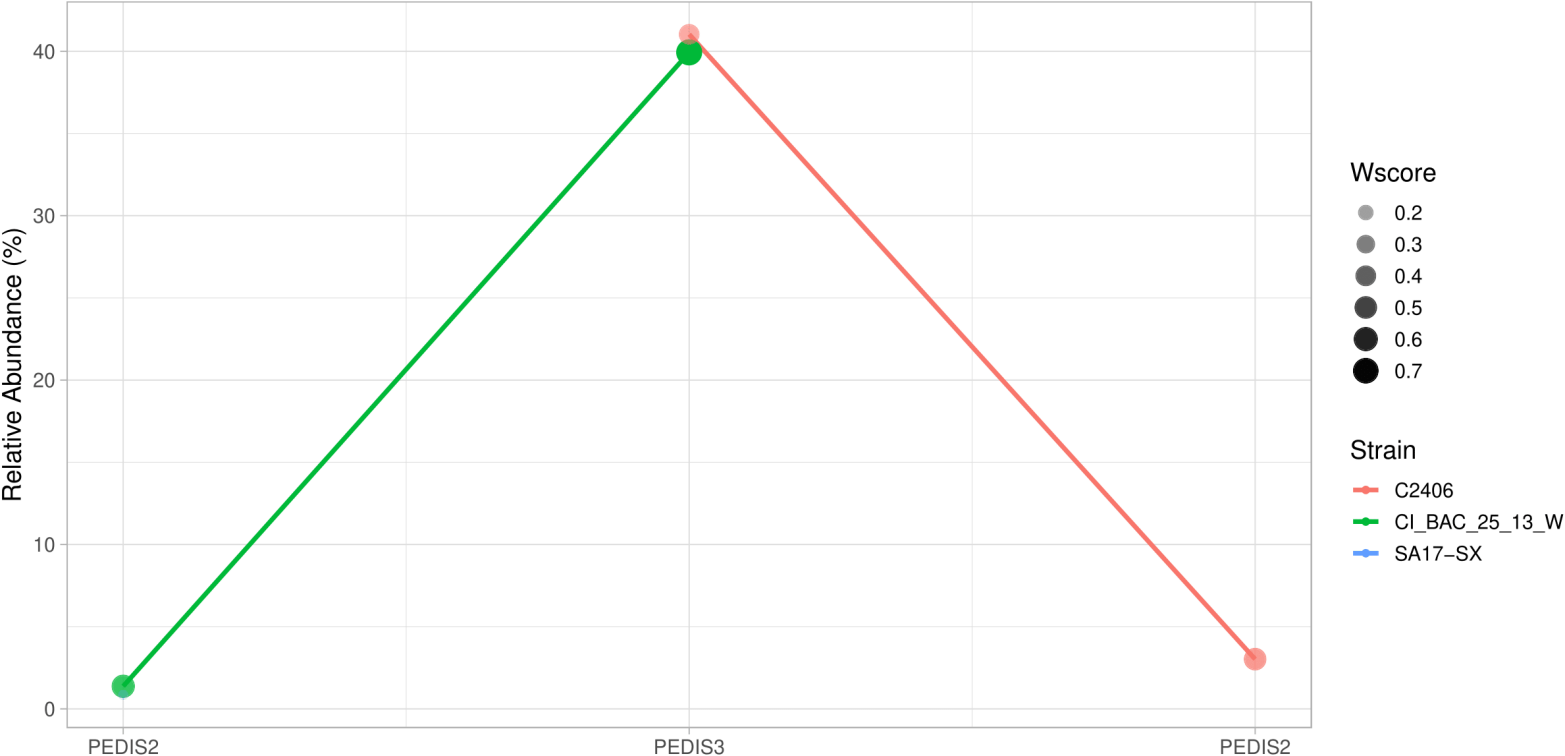
Temporal changes in *S. aureus* strain abundance identified within P2. Wscore is indicative of the confidence of predicted strain match by StrainGST. Wscore for each strain within each sample is indicated by the size and color of each point.

Strain analysis was performed using StrainGE with custom-built databases tailored to the predicted pathogens identified in the wounds. Strain variation was only observed in P2 with changing infection severity (PEDIS 2 > PEDIS 3 > PEDIS 2), with the shift to moderate infection being associated with a bloom of *S. aureus* (**Figure 5**). StrainGST identified three strains in P2 including CI_BAC_25_13_W, C2406 and SA17-SX (**Figure 4**). Increasing infection severity coincided with an increase in abundance of CI_BAC_25_13_W (1.4% to 39%, high confidence, wscore = 0.716) and the emergence of C2406 at 41% abundance (moderate confidence, wscore = 0.396). Following treatment with Augmentin Duo Forte, infection severity decreased which coincided with the clearance of CI_BAC_25_13_W and a decreased abundance of C2406 to 3% (wscore = 0.498). StrainGR confirmed that these strains were identical across patient samples based on common callable genome (>0.5%), Jaccard gap similarity (>0.95%) and ANI (>99.95%), indicating that the observed dynamics reflect expansion and reduction of the same strains rather than the introduction of new strains.

## Discussion

In this study, the microbiome of DRFUs were longitudinally profiled to identify within-patient microbial shifts associated with infection onset and infection resolution. Our results showed that: (1) Microbiomes are highly heterogenous between patients which highlights issues with cross-sectional studies; (2) Virulent pathogens are colonizing non-infected DRFUs; (3) in non-infected DRFUs colonized by gram-positive pathogens, infection onset is associated with a shift toward increased abundance of obligate anaerobes and a reduction in gram-positive pathogens; and (4) Anaerobe abundance decreases following infection resolution and/or reduction of infection severity.

We explored the microbiome of 5 patients who transitioned from a non-infected to infected state and 1 patient with changing infection severity as determined by clinical observation. Samples were collected before, during and after infection. Shotgun metagenomics was utilized to analyze within-patient microbial shifts using marker-based tools (metaphlan3), metagenome assembly/binning and strain level analysis. Diversity analysis revealed that patient variability explained the majority of variance observed between samples, highlighting a common problem associated with microbiome studies that are non-longitudinal by design (Flores et al., 2014). Therefore, subsequent analysis was completed either within patients or by including patient identity as a random effect. Linear mixed models identified a significant difference in the beta diversity between infected and non-infected wounds and there was no difference in alpha diversity using the Richness, Simpson and Shannon indices.

Analysis of the most abundant species and binned genomes identified pathogens commonly observed in DRFIs, namely gram-positive pathogens including *S. aureus*, *E. faecalis* and *Streptococcus* spp. and gram-negative species including *E. hormaechei* and *P. aeruginosa*. Binned genomes of these species were identified to carry a significant number of virulence factors. Importantly, in each patient the most abundant virulent bin identified by metagenomics had a corresponding isolate cultured by the hospital’s pathology department, providing independent validation of the metagenomic findings and demonstrating concordance between molecular and culture-based approaches. This suggests that symptoms associated with acute infection are driven primarily by these pathogens. Interestingly, virulent pathogens were identified within baseline wounds with no clinical signs of infection, with time between sampling non-infected and infected wounds ranging from 2 to 30 weeks. In P3, P4, P5 and P6, we observed a decrease in the abundance of gram-positive virulent pathogens upon infection onset. Additionally, at the time of baseline sampling P3 was classified as a stable wound and P4 and P5 were classified as healing, despite the presence of pathogens. These findings support a study by Gardner *et. al.*, which identified no association between the presence of potential pathogens or microbial load on infection onset and wound outcomes in DRFUs (Gardner et al., 2014). This further suggests that colonization of DRFUs by pathogens carrying multiple VFs alone is not sufficient to cause infection, and alludes to other factors/conditions being required for the transition (from colonization and infection) to occur.

Interestingly, for most patients we observed an increase in the abundance of obligate anaerobic species with infection onset. The role of anaerobic species in chronic wounds has been underestimated due to the difficulty in their cultivation and identification (Uberoi et al., 2024). The adoption of molecular sequencing techniques in recent years has begun to highlight their ubiquity within DRFUs with studies now reporting their presence in 78-100% of wounds, with the abundance of anaerobes being positively correlated with non-healing wounds, infection severity and risk of amputation (Coluccio et al., 2024). In the data presented here, we identified that anaerobe abundance increased in 4/5 patients during infection which was then reversed upon infection resolution or downgrade of infection severity. The dominant anaerobes identified were unique to each patient, however several genera were identified across patients including *Finegoldia* spp, *Prevotella* spp, *Porphyromonas* spp, *Peptoniphuilus* spp, *Peptostreptococcuss* spp and *Anaerococcuss* spp.

Of these genera, *Prevotella* spp and *Porphyromonas* spp are the most studied and have been shown to be pro-inflammatory. Specifically, studies on *P. gingivalis* have identified its ability to promote certain features of inflammation while simultaneously supressing antimicrobial processes. In doing so, *P. gingivalis* obtains nutrients via inflammation-derived tissue damage and avoids immune elimination by supressing the innate immune response (Maekawa et al., 2014; Olsen et al., 2017; Wang et al., 2010). *Prevotella* spp can elicit a similar response and have been shown to activate T helper type 17 cells and promotes neutrophil infiltration, which they can subsequently kill, protecting them and neighbouring bacteria from clearance (Matsui et al., 2014).

Fermentation products have also been shown to alter both the wound microenvironment and microbiome. Anaerobic bacteria produce short chain fatty acids (SCFAs) as a biproduct of fermentation, several of which have been identified in chronic wounds including propionate and butyrate (Junka et al., 2017). The impact of SCFAs on the wound microbiota is not well understood, however evidence suggests that fermentation products can modulate the activity of different bacteria. Specifically, mucin fermentation has been shown to promote the expression of virulence factors of *S. aureus* in chronic rhinosinusitis infections. Furthermore, the anti-inflammatory effects of SCFAs are well known by modulating cytokine production, chemotaxis, phagocytic capacity and the generation of reactive oxygen species (ROS) (Correa-Oliveira et al., 2016). It is therefore reasonable to speculate that an increased abundance of anaerobic bacteria would result in an increase in localized SCFAs within the wound. This may then suppress the immune response and allow virulent pathogens already present within the wound to drive acute infection and cause tissue damage. It is important to note that this trend involving anaerobic species was not observed in P1, who had an infection likely driven by *E. hormechei* (gram-negative rod) rather than gram-positive cocci. While it is not possible to make any conclusions on this observation due to restricted numbers (n=1), this result may indicate alternative infection pathways utilized by gram-negative pathogens that may not correlate with the activity of obligate anaerobes.

It is also critical to note the importance of hypoxia in relation to DRFUs. Under normal conditions the host response to hypoxia is regulated by HIF-1, a transiently expressed transcriptional regulator that controls a variety of cellular processes, including those essential for tissue repair, wound healing and angiogenesis (Zhu et al., 2024). Importantly, HIF-1 activation is observed during bacterial infections through both oxygen-dependent and independent mechanisms, and its activation is protective against localized infection as observed through *in vitro* and *in vivo* studies (Devraj et al., 2017). Several studies have identified that phagocytosis by macrophages is HIF-1-dependent in hypoxic environments and that neutrophils are protected from apoptosis via HIF-1-dependent NF-κB activation (Anand et al., 2007; Walmsley et al., 2005). In diabetic wounds, HIF-1 signaling is impaired due to hyperglycemia which results in increased degradation of HIF-1α by prolyl hydroxylase domain-containing proteins (PHDs) (Catrina and Zheng, 2016; Mahon et al., 2001).

Combined, the findings presented here support a potential model in which the wound microbiome shifts from colonization to infection through a series of host-microbial interactions, potentially driven by hypoxia. As seen in our data, baseline wounds are colonized by pathogenic species, yet remain clinically stable, potentially due to residual immune responses which is sufficient to maintain microbial control, with bacteria possibly present as biofilm which are ubiquitous in chronic wounds (James et al., 2008). Acute or progressive ischemic events can then reduce tissue oxygen saturation, which combined with the impairment of HIF-1 signaling, facilitates an anaerobic bloom and restructuring of the microbial community and the wound microenvironment. Hypoxia, reduced HIF-1 signaling and the proliferation of anaerobes likely contributes to immune modulation which creates an environment that favors pathogen activation. Under these conditions, previously colonizing pathogens can transition to an invasive phenotype resulting in acute infection. This anaerobe associated trajectory explains the trends observed in wounds associated with gram-positive pathogens, whereas trends observed in P1 may indicate a distinct gram-negative pathway which is more consistent with direct virulence driven invasion, independent of anaerobic synergy. These models are supported by observations from Hunter et al., which utilized topical oxygenation therapy (TOT) on chronic DRFUs. They identified that 5/6 wounds healed following TOT and had an associated shift in the microbiome to be dominated by aerobes and facultative anaerobes, whereas anaerobic species persisted in 1 patient who did not heal (Hunter et al., 2020).

There are several limitations to this study. First, the cohort size was small (n=6) which limits the power of our findings and restricts the interpretation of the results discussed to being speculative rather than conclusive, while also highlighting the difficulty in capturing infective events longitudinally. Second, not all patients had complete longitudinal sampling across baseline, infection onset and resolution, limiting the ability to fully capture temporal dynamics for every individual. Third, despite host DNA depletion, the proportion of microbial reads was relatively low for some samples, which may have impacted the detection of low-abundance species, the ability to bin genomes and achieve strain-level resolution. Fourth, all patients received systemic antibiotic therapy following infection diagnosis which may have influenced microbial composition at post-infection resolution timepoints. However, with the exception of P2 who was receiving oral Cephalexin prior to enrollment, no patients were receiving antibiotic therapy at baseline or prior to infection onset. The primary finding of an increase in obligate anaerobe abundance at infection onset therefore reflects a transition occurring in the absence of antibiotic pressure in 5/6 patients and is unlikely to be attributable to antibiotic-induced microbial changes. Finally, important clinical data is required to confirm the proposed model of infection, such as longitudinal tracking of tissue oxygen saturation, which is not routinely performed, as well as monitoring of host markers such as HIF-1 and genes which it regulates.

Despite these limitations, our study provides novel insights into within patient microbial shifts during the onset and resolution of DRFIs and highlights the utility of longitudinal metagenomic approaches for understanding wound microbiology. Future studies with larger cohorts and improved host depletion methods will be required to validate these findings and refine our understanding of DRFI pathology. Additional host data will also be required to identify associations between infection, anaerobe abundance and host immune factors/wound oxygen saturation.

## Conclusion

To our knowledge, this is the first study to track longitudinal changes in the microbiome within patients as they progress from non-infected DRFUs to DRFIs. By utilizing shotgun metagenomic sequencing we were able to capture temporal microbial shifts, including the emergence and decline of specific pathogens that are not observable in cross-sectional studies. Our results identified that infection onset is associated with anaerobic blooms and in some cases, the decreased abundance of specific pathogens, potentially highlighting the role of transient shifts in wound oxygen saturation on the microbiome and subsequent infection onset. We also found that virulent pathogens colonize clinically non-infected wounds and that changes in infection severity are associated with shifts in strain dynamics. Combined, these findings; (1) highlight the importance of within patient microbial dynamics in understanding DRFI pathogenesis; (2) highlight the potential importance of monitoring wound oxygen saturation throughout clinical care as a potential marker for infection risk, which is not currently performed as standard of care; and (3) may support the use of therapies such as TOT to maintain stable levels of oxygenation within DRFUs, or PHD inhibitors to improve HIF-1 dependent responses to hypoxia.

## Data Availability

The datasets presented in this study can be obtained from the Sequence Read Archive project ID PRJNA1371406.

## Supplementary material

Supplementary Figure 1 Total reads generated and reads remaining after host depletion.

Supplementary Figure 2 Longitudinal changes in Bray-Curtis dissimilarity relative to the baseline sample in each patient. An increase in distance indicates a change in diversity away from baseline and a decrease indicates a change in diversity toward baseline.

Supplementary Data Sheet 1 Genome bins with associated virulence factor and antimicrobial resistance gene counts.

Supplementary Data Sheet 2 Full Metaphlan3 taxonomic classifications.
