# Supplementary material for: Anaerobe-associated microbial shifts at infection onset in diabetes-related foot ulcers revealed by longitudinal metagenomics
Source: Front Cell Infect Microbiol. 2026 Jun 2;16:1812721. doi: 10.3389/fcimb.2026.1812721 (PMC13269009; doi:10.3389/fcimb.2026.1812721)
Supplement: Supplementary Figure 1 — Total reads generated and reads remaining after host depletion. [file Table1.docx]

Supplementary Figures
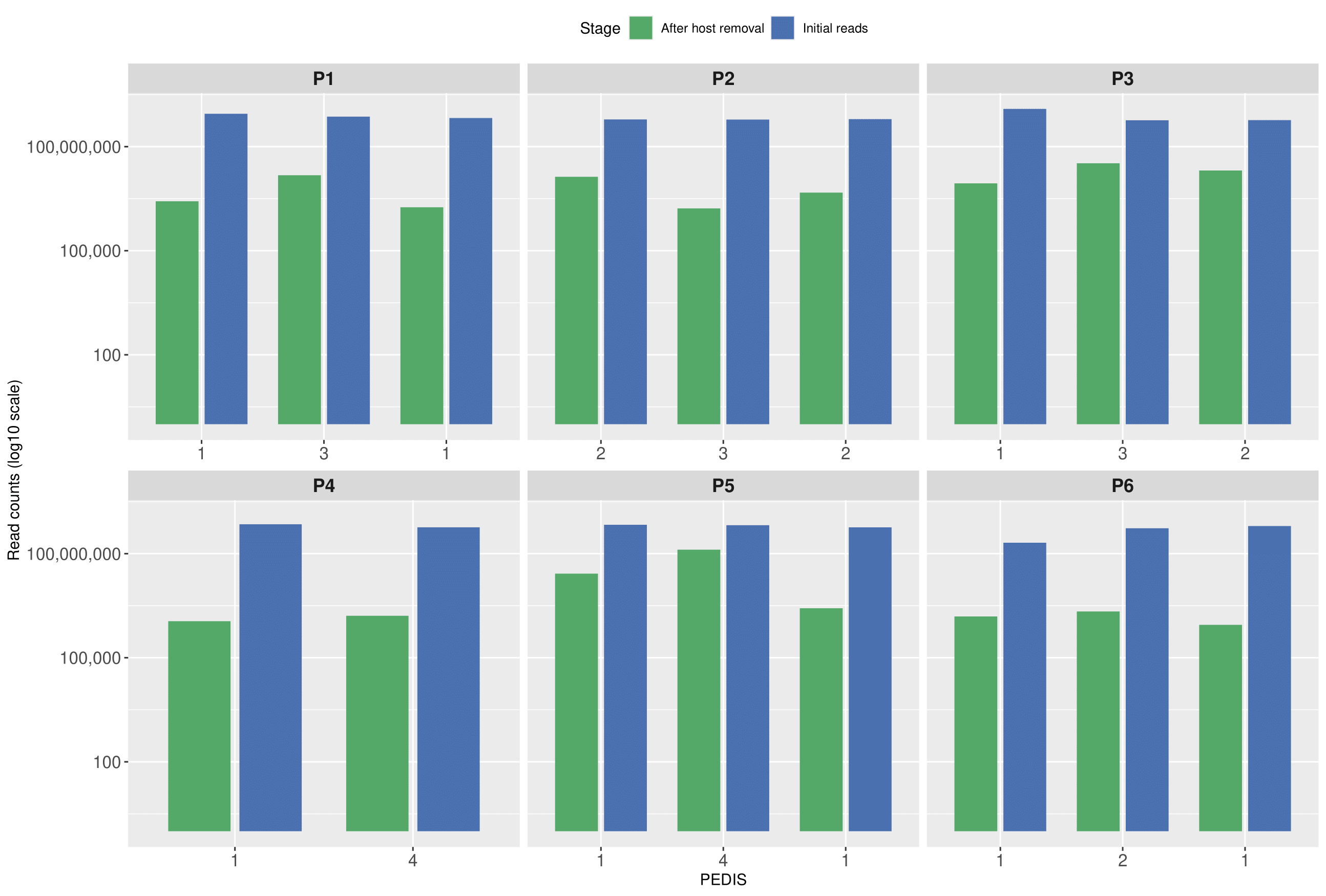
e

Supplementary Figure 1: Total reads generated and reads remaining after host depletion


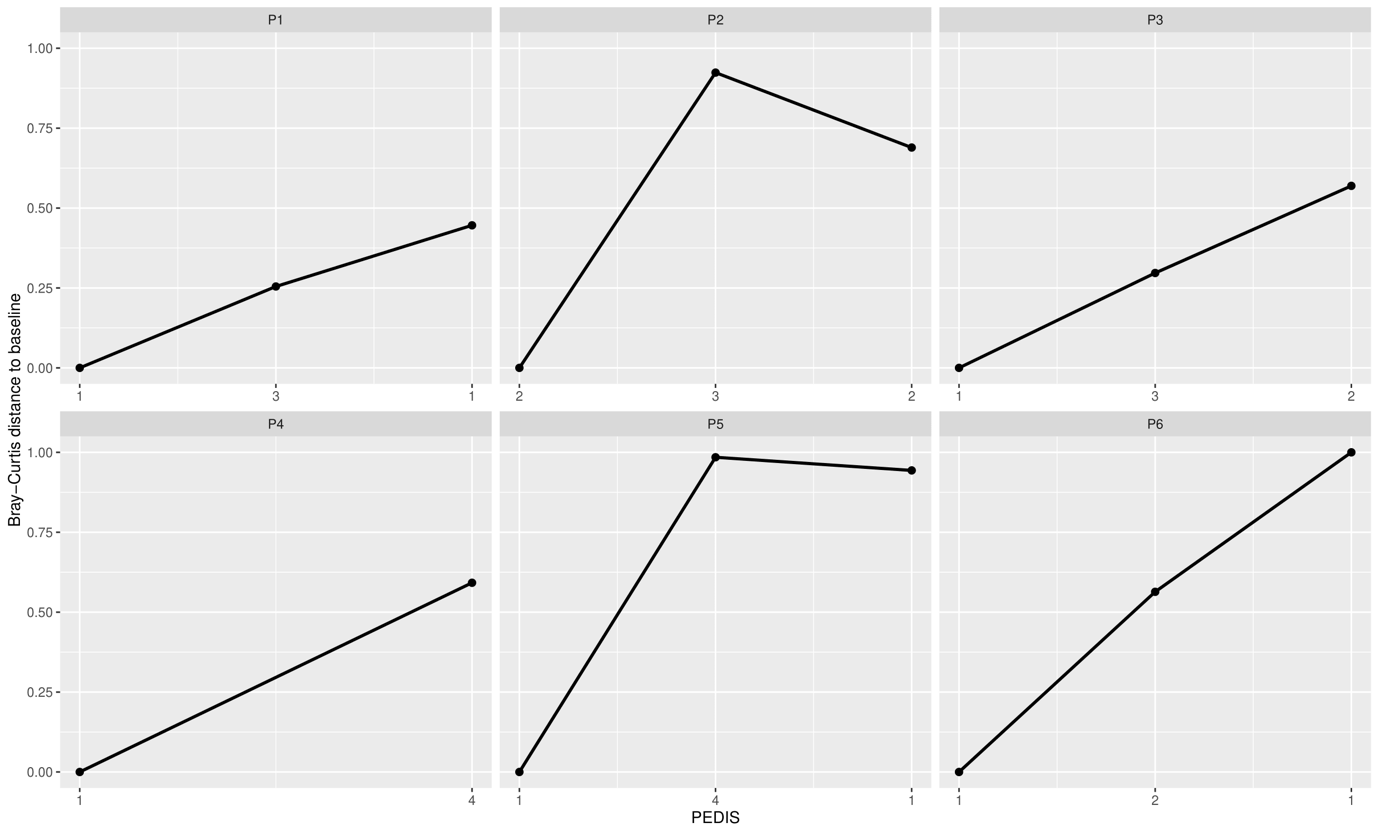


Supplementary Figure 2: Longitudinal changes in bray-curtis dissimilarity relative to baseline sample in each patient. An increase in distance indicates a change in diversity away from baseline and a decrease indicates a change in diversity towards baseline
